# Correlation between variation in ambient temperature and presentation to the emergency department for psychiatric disorders: results from an eight-year observational study

**DOI:** 10.1192/j.eurpsy.2026.11103

**Published:** 2026-07-31

**Authors:** N. Rizzo Pesci, S. Peracchia, G. Maina, G. Rosso

**Affiliations:** 1 Department of Neurosciences “Rita Levi Montalcini”, University of Turin, Turin; 2 Unit of Psychiatry, San Luigi Gonzaga University Hospital, Orbassano, Turin, Italy

## Abstract

**Introduction:**

Climate change is increasingly recognized as a public health emergency. Rising temperatures and extreme weather events affect morbidity rates and the use of emergency services. In particular, high temperatures have been associated with increased emergency department (ED) visits and hospital admissions for mental health disorders, as well as with a rise in suicide attempts. Despite growing evidence on this topic, the available data remain heterogeneous and inconsistent.

**Objectives:**

The aim of this study is to assess the correlation between increasing ambient temperature and emergency department visits for mental health disorders.

**Methods:**

This is a cross-sectional observational study. ED presentations for psychiatric disorder over an eight-year period were recorded and aggregated according to the standardized weekly index (ISO weeks 1–53). For each ISO week, thermal anomalies were calculated relative to the average weekly temperature across the observation period. Correlation between temperature anomaly and ED visits within the same weekly window was then analyzed.

**Results:**

A significant association was found between temperature anomaly and the number of psychiatric ED visits (Pearson r = 0.110, p = 0.0369; Spearman ρ = 0.107, p = 0.0416).

**Conclusions:**

These findings support the hypothesis that rising ambient temperatures impact on the course of psychiatric disorders. This highlights the potential of temperature as an indicator to optimize patient follow-up and allocation of resources in the emergency settings. Further analyses will be conducted by stratifying patients according to the reason for ED presentation and by controlling for potential confounding factors. Enrichment of the model with additional variables (humidity, weather conditions, pollutants, holidays), and increased time resolution, will further contribute to the understanding of the mechanisms underlying the observed correlation. This could foster the development of forecasting systems to dynamically modulate patient follow-up, hospital staffing and bed management.

**Disclosure of Interest:**

None Declared

